# Dynamic Adsorption of Sulfamethoxazole from Aqueous Solution by Lignite Activated Coke

**DOI:** 10.3390/ma13071785

**Published:** 2020-04-10

**Authors:** Haiyan Li, Juan He, Kaiyu Chen, Zhou Shi, Mengnan Li, Pengpeng Guo, Liyuan Wu

**Affiliations:** 1 Beijing Engineering Research Center of Sustainable Urban Sewage System Construction and Risk Control, Beijing University of Civil Engineering and Architecture, Beijing 100044, China; Lihaiyan@bucea.edu.cn (H.L.); louyounan@163.com (J.H.); Chenkaiyuxiao@163.com (K.C.); shizhou2016@163.com (Z.S.); lmn970109@163.com (M.L.); gzpjustice@163.com (P.G.); 2 Beijing Advanced Innovation Center for Future Urban Design, Beijing 100044, China

**Keywords:** lignite activated coke, sulfamethoxazole, dynamic, adsorption, penetration curve, modeling

## Abstract

In this paper, lignite activated coke was used as adsorbent for dynamic column adsorption experiments to remove sulfamethoxazole from aqueous solution. The effects of column height, flow rate, initial concentration, pH and humic acids concentration on the dynamic adsorption penetration curve and mass transfer zone length were investigated. Results showed penetration time would be prolonged significantly by increasing column height, while inhibited by the increasement of initial concentration and flow rate. Thomas and Yoon-Nelson model and the Adams-Bohart model were used to elucidate the adsorption mechanism, high coefficients of R^2^ > 0.95 were obtained in Thomas model for most of the adsorption entries, which revealed that the adsorption rate could probably be dominated by mass transfer at the interface. The average change rates of mass transfer zone length to the changes of each parameters, such as initial concentration, the column height, the flow rate and pH, were 0.0003, 0.6474, 0.0076, 0.0073 and 0.0191 respectively, revealed that column height may play a vital role in dynamic column adsorption efficiency. These findings suggested that lignite activated coke can effectively remove sulfamethoxazole contaminants from wastewater in practice.

## 1. Introduction

Pharmaceuticals and personal care products (PPCPs) have become emerging pollutants due to the large amount of human consumption and usage [1,2,3,4]. Relating to the properties of high polarity and low volatility, PPCPs tend to be distributed and migrated to the environment through water phase transfer and food chain diffusions [5,6]. At present, many PPCPs pollutants have been detected in surface water, groundwater, drinking water and sewage, in the level of ng/L to μg/L, which will have potentially toxicological effects on the aquatic organisms [6]. Besides, the accumulation of these chemicals through the food chain may be harmful to human health [7]. Thus, it is necessary to develop effective treatment options to reduce their release into the environment. Up to now, various methods to remove PPCPs from wastewater have been developed, including photocatalysis [8,9], advanced oxidation [10,11], electrocatalysis [12,13], adsorption [14,15] and so on. 

Sulfamethoxazole is a typical broad-spectrum antibiotic, which has been used in large quantities due to its inhibiting ability towards bacteria sensitivity [16]. Many methods, such as photocatalysis, advanced and fenton oxidation technology can effectively removal sulfamethoxazole from aqueous solution but the complicated operations, high cost and safety concerns has limited their application [14,16,17,18,19,20]. Therefore, the adsorption method is often used in industrial wastewater treatment due to the convenient operation in the advanced treatment stage. Different materials, such as carbon materials [21,22,23], composite metal materials [24,25,26], graphene oxide [27,28], metal-organic frameworks (MOFs) [29], resins [30] and so on have been reported with considerable adsorption capacity for sulfamethoxazole. However, considering practical use for wastewater treatment, economic materials with good mechanical strength, high adsorption capacity and fast removal efficiency are still being researched for sulfamethoxazole removal from aqueous solution. Lignite activated cokes (abbreviated as LACs), which are produced from carbonaceous materials, have been widely used in water treatment in recent years due to their benefits of high mechanical strength, cheapness, macroporous and mesoporous structures and oxygen-containing organic functional groups [31,32,33]. These have attracted extensive attention to LACs in the field of wastewater treatment in recent years. In this paper, the dynamic adsorption behavior of LACs towards sulfamethoxazole in aqueous solution was investigated. 

The effects of column height, initial concentration, flow rate, humic acids and pH on dynamic adsorption behavior were comparatively analyzed to determine the optimized condition for dynamic adsorption. The models of Thomas, Yoon-Nelson and Adams-Bohart were fitted to experiment data and the Thomas has high correlation coefficients. The homogeneous surface diffusion model (HDSM) was fitted to a breakthrough curve and obtained the surface diffusion constant.

## 2. Materials and Methods 

### 2.1. Materials, Reagents and Tests

Commercial LACs (1–2 mm) with a specific surface area of 790 m^2^ g^−1^ was obtained from Beijing Guodian Futong Technology Co., Ltd. (Beijing, China) and purified as follows [34]. Two grams of LACs was poured into a beaker containing 500 mL deionized water, boiled for 30 min, washed with deionized water for several times and dried at 105 °C for 24 h. Sulfamethoxazole (analytical grade) was purchased from Macleans Corporation (>99%, Shanghai, China). Humic acids, NaOH, HCl and NaCl were purchased from Sinopharm (Shanghai, China). These chemicals were used directly without purification.

The concentration of sulfamethoxazole in aqueous solution was measured by Acquity UPLC-H, with a C18 analytical column (1.7 µm × 2.1 mm × 50 mm) (ACQUITYUPLC, Waters, Milford, MA, USA), mobile phase (0.01% acetic acid solution: methanol = 30:70), flow rate (0.35 mL/min), column temperature 303 K, λ = 265 nm and the retention time was 0.7 min.

### 2.2. Equipment and Conditions of Column Experiments 

#### 2.2.1. Column Experimental Device

**Figure 1:**
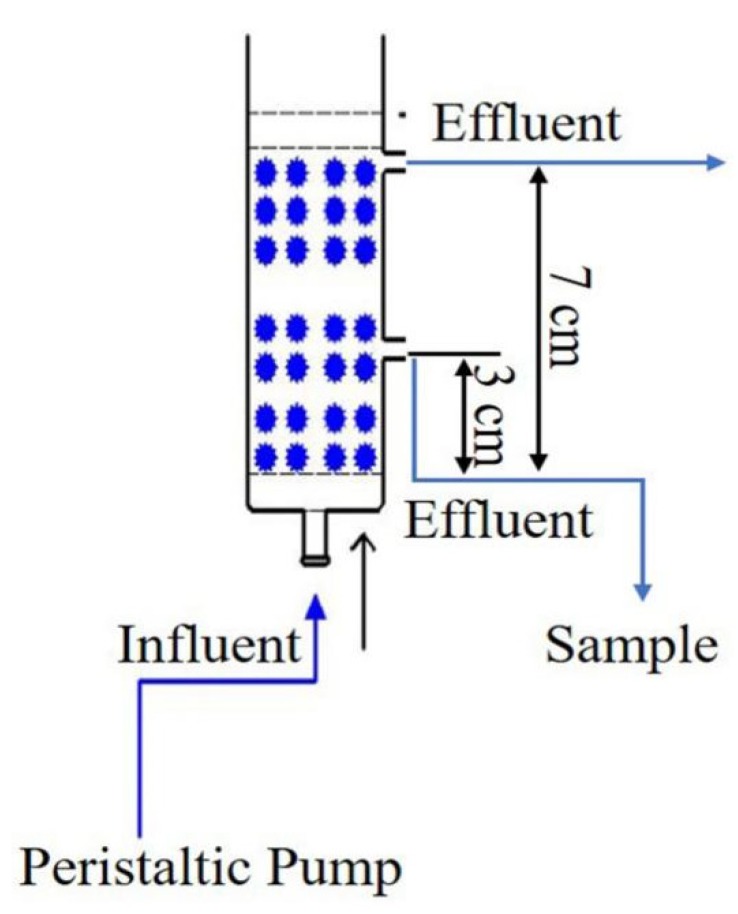
The experimental device of column adsorption.

All the dynamic adsorption experiments were operated in a series of abbreviated wet packing glass columns, with an inner diameter of 2.5 cm, as shown in Figure 1. For each column, 0.5 cm glass beads, 3 cm LACs (with particle size of 100 mesh) and 0.5 cm glass beads were successively filled into the column, pressed tightly and uniformly. Prior to the experiment, the deionized water was inflowed into the column completely to exclude the air in the porosity of cokes until the pH of effluent arrived neutral, thus to maintain the stability of pore structures inside the adsorption column. ρ_0_ is the loading density of activated coke in the column (g/cm^3^). The aqueous solution of sulfamethoxazole was loaded into the column through the inlet at the bottom of the reactor and suctioned by a peristaltic pump under a steady flow. Samples were taken from the outlet at the fixed time intervals. Except for the column height experiment, the other experiment samples were received from outlets located at a column height of 3 cm. The concentrations of sulfamethoxazole in the sampling solution were tested by UPLC with a retention time of 0.7 min. With this device, the dynamic adsorption behaviors under different operation factors such as pollutant concentration, flow rate, pH and packing column height were investigated.

#### 2.2.2. Column Experiments

For the column adsorption experiments, the sulfamethoxazole solution (ion strength of 10 mmol/L NaCl) was pumped into the column from bottom to top by a peristaltic pump. The effect of different initial sulfamethoxazole concentrations (35 mg/L “vs.” 75 mg/L), column heights (3 cm “vs.” 7 cm), inlet flow rates (3 mL/min “vs.” 5 mL/min), solution pH (4, 6.5 and 8) and humic acids concentration (0, 0.1, 1 and 10 mg/L) on breakthrough curves were studied. 

In the pH effect experiments, sulfamethoxazole solutions (35 mg/L) with initial pH values of 4, 6.5 and 8 were prepared, respectively. Aqueous HCl (0.10 mol/L) or NaOH (0.10 mol/L) were used to adjust the pH. Two outlets were designed in the column with a height of 3 cm and 7 cm respectively. In the column experiments with column height of 7 cm, the lower outlet at 3 cm was sealed. At certain intervals, in eluate, the concentration of the sulfamethoxazole was determined. 

Herein, the time with the sampling concentration reaching 10% and 95% of the initial concentration were defined as the adsorption penetration point (*t_b_*, min) and the adsorption penetration end time (*t_e_*, min). The length (*H*, cm) of the mass transfer zone of the adsorption column was calculated based on the adsorption penetration point time *t_b_* and the adsorption penetration end time *t_e_* as follows (1):

(1) $$H=\frac{C_{0}Q}{q\rho_{0}A}\left(t_{e}-t_{b}\right)$$

*Q* represents the flow rate (mL/min); *C*_0_ is the inlet sulfamethoxazole concentration (mg/L); *q* is the amount adsorption capacity (mg/g) of sulfamethoxazole by unit activated coke; *ρ*_0_ is the loading density of activated coke in the column (g/cm^3^). *A* is the cross-sectional area of the adsorption column (cm^2^).

The adsorption capacity *q* is calculated according to Equation (2) [15,35]:

(2) $$q=\left(C_{0}-C_{e}\right)\frac{V}{M}$$

Among them, *C*_0_ and *C_e_* were determined by UPLC with a retention time of 0.7 min. *q* is the dynamic saturated adsorption amount (mg/g); *C*_0_ is the inlet concentration of sulfamethoxazole (mg/L); *C_e_* is the outlet concentration of sulfamethoxazole; *V* (L) is the solution volume; *M* is the mass weight of the adsorbents (g).

## 3. Results and Discussion

### 3.1. Adsorption Performance of Sulfamethoxazole on LACs

#### 3.1.1. Effect of Initial Concentration 

**Figure 2:**
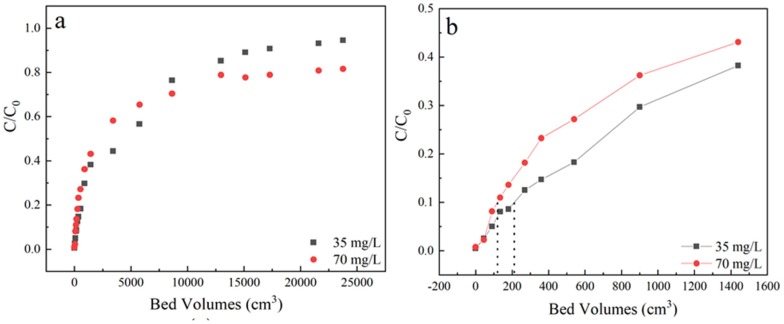
(**a**) The effect of different concentration of sulfamethoxazole adsorption breakthrough curves; (**b**) Partially enlarged screening from 0 to 1600 bed volume. Experiment condition: flow rate of 3 mL/min, column height of 3 cm, pH 6.5, sulfamethoxazole concentration of 35 and 70 mg/L, respectively.

The breakthrough curves of sulfamethoxazole adsorption performance on LACs were described from a series of column adsorption entries to evaluate the enrichment capacity of LACs. At a fixed column height of 3 cm, a flow rate of 3 mL/min, pH 6.5, column adsorption under initial sulfamethoxazole concentration of 35 mg/L and 70 mg/L were comparatively studied. The samples were received from the outlet located at a column height of 3 cm. As shown in Figure 2 and Table 1, with the increase of initial concentration, the bed volume decreased from 210 to 120 cm^3^. 

The time required to reach 50% of initial concentration was obviously extended from 12 h to 25 h, which may be due to that high initial concentration, increased the sulfamethoxazole concentration difference between activated coke and solutions, making the breakthrough curve slope steeper [36]. On the other hand, it was clear that the time required to reach saturation decreased with the increasing of the initial concentration, as the diffusion rate is controlled by the concentration gradient. As the initial concentration of pollutants increased, the bed utilization rate decreased. Besides, the adsorption penetration end time in the system of 70 mg/L could not be reached, the C/C_0_ after 140 h was close to 0.8 and arrived in equilibrium, which indicated that a certain retention effect may exist in the liquid flow system of the adsorption column under high sulfamethoxazole initial concentration.

#### 3.1.2. Effect of Column Height 

**Figure 3:**
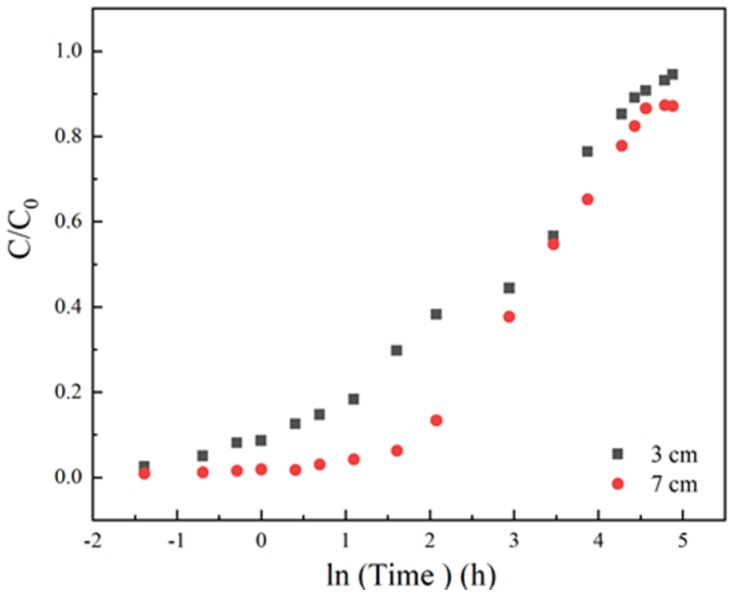
The adsorption effect of different column height on breakthrough curves, Experiment condition: sulfamethoxazole concentration of 35 mg/L, flow rate of 3 mL/min, pH 6.5, column height 3, 7 cm, respectively.

At a fixed flow rate of 3 mL/min, initial sulfamethoxazole concentration of 35 mg/L and pH 6.5, column adsorption under different column heights of 3 cm and 7 cm were studied to assess the effect of column height on the dynamic adsorption. From Figure 3 and Table 1, it was observed that column height had a positive relationship with the bed adsorption capacity. Under a high column height of 7 cm, the bed volume could be extended remarkably from 210 to 395 cm^3^. Besides, the curve slope became smooth under a higher column length. Undoubtedly, the amounts of LACs increased, the adsorption capacity could be enhanced, and high breakthrough time gave better intraparticle diffusion phenomena. Some references have mentioned that carbon-based materials with high mesopores, large pore volumes and medium specific surface area have the best adsorption effect on sulfamethoxazole [32,37]. LACs contained a number of macropore and mesoporous structures, attributed not only to the increasing of the specific surface area but also enhancing the sulfamethoxazole removal by increasing the spread of contaminant and a capacity of the sorbent material [34]. Moreover, LACs contain many oxygen-containing functional groups, such as phenolic groups, which could provide abundant binding sites for sulfamethoxazole through hydrogen-bonding interactions. The curves shape noted for 3 cm was more upright than 7 cm, this might be because a larger mass transfer region has formed in the longer column, which retarded the arrival of penetration time.

#### 3.1.3. Effect of Flow Rate 

**Figure 4:**
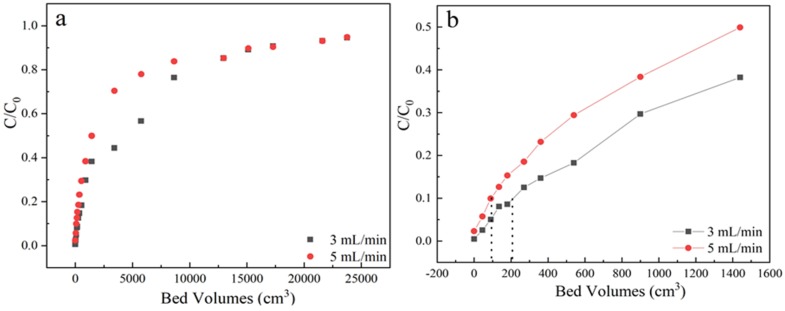
(**a**) The adsorption effect of different flow rate on breakthrough curves; (**b**) Partially enlarged screening from 0 to 1600 bed volume. Experiment condition: sulfamethoxazole concentration of 35 mg/L, pH 6.5, column height of 3 cm, flow rate of 3, 5 mL/min, respectively.

Flow rate is an important parameter for industrial-scale wastewater treatment. The samples were received from the outlet located at a column height of 3 cm, sulfamethoxazole concentration of 35 mg/L at pH 6.5, the adsorption flow rates were varied from 3 mL/min to 5 mL/min, the corresponding dynamic adsorption breakthrough curves were compared in Figure 4 and Table 1. It could be found that the steepness increased with the flow rate, resulting in an earlier breakthrough point volume under 5 mL/min. 

This is because that low flow rate can offer enough time for intra-particle diffusion of pollutants at the interface of adsorbents as well as a binding interaction between sulfamethoxazole and functional groups of LACs. And the flow rate will affect the external film diffusion but not the surface diffusion. Contrarily, high flow rate of 5 mL/min could easily cause the decrease of mass transfer resistance, then the breakpoint time and saturation were quickly reached. Besides, fast flow rate would also reduce the utilization efficiency of the fixed bed before reached to saturation.

#### 3.1.4. Effect of pH

In the actual wastewater treatment process, pH factor will affect the effectiveness of the adsorbent to remove pollutants. So, it is necessary to consider the pH effect on wastewater treatment. Herein, effect of pH was executed at 4, 6.5 and 8 under the condition of 35 mg/L sulfamethoxazole concentration, 3 mL/min flow rate and the samples were received from the outlets, located at a column height of 3 cm.

**Figure 5:**
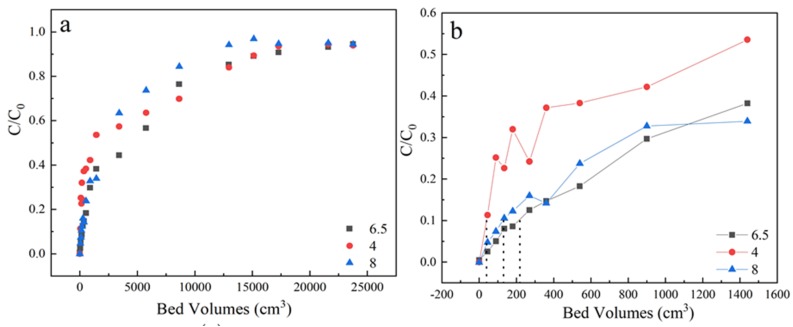
(**a**) The adsorption effect of different pH on breakthrough curves, (**b**) Partially enlarged screening from 0 to 1600 bed volume. Experiment condition: flow rate of 3 mL/min, column height of 3 cm, sulfamethoxazole concentration of 35 mg/L, pH 4, 6.5, 8 respectively.

As shown in Figure 5 and Table 1, the best adsorption performance was obtained at pH 6.5. The pH_pzc_ of activated coke was 6.5 and the p_Ka_ of sulfamethoxazole was 5 [38]. At pH 4, electrostatic interaction contributes to the major adsorption mechanism due to the negatively charged sulfamethoxazole species and the positively charged surface of LACs. At pH 6.5, the sulfamethoxazole species were negatively charged while the surface of LACs was nearly uncharged, the hydrogen-bonding interaction played an important role during adsorption because the phenolic groups of LACs and the N, O atoms of sulfamethoxazole species. Similar observations were also reported in diclofenac sodium adsorption on oxidized activated carbon [39]. At pH 8.0, the sulfamethoxazole species and the surface of LACs were both negatively charged, the electrostatic repulsion could inhibit the sulfamethoxazole adsorption, thus a lowered penetration time was obtained.

#### 3.1.5. Effect of Humic Acids

**Figure 6:**
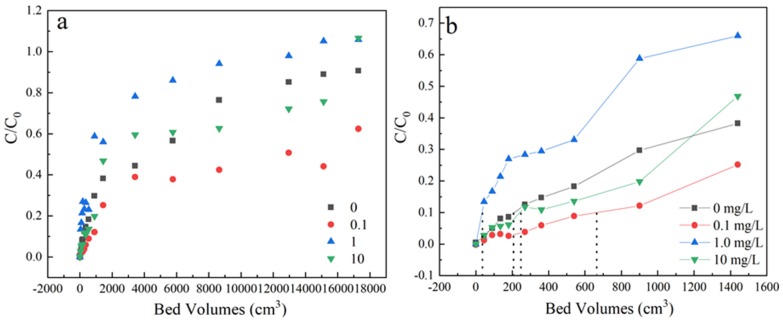
(**a**) The adsorption effect of different concentration of human acids on breakthrough curves, (**b**) Partially enlarged screening from 0 to 1600 bed volume. Experiment condition: flow rate of 3 mL/min, column height of 3 cm, sulfamethoxazole concentration of 35 mg/L, pH 6.5, humic acids concentration= 0, 0.1, 1, 10 mg/L, respectively.

The effect of humic acids was executed at 0, 0.1, 1 and 10 mg/L under the condition of 35 mg/L sulfamethoxazole concentration, 3 mL/min flow rate and the samples were received from the outlets, located at a column height of 3 cm. As shown in Figure 6 and Table 1, the penetration time increase slowly during 0–0.1 mg/L and decreased deeply when organic concentration increased to 1 mg/L. When increasing the concentration of organic matter to 10 mg/L, the adsorption ability increased a little. The slopes of penetration curves under different humic acids concentration follows in the order of 1 > 0 > 10 > 0.1 mg/L. These results illustrated that the existence of humic acids would affect the dynamic adsorption behavior.

### 3.2. Breakthrough Curves Models Analysis

To elucidate the adsorption mechanism of sulfamethoxazole on LACs, the Thomas model, Yoon-Nelson and Adams-Bohart model were used to predict the performance and parameters of the fixed-bed column. The Thomas model is derived depending upon second-order kinetics and assumed that the sorption is dominated by mass transfer at the interface not the chemical reaction [40]. The mathematical form is as follows:

(3) $$\ln\left(\frac{C_{0}}{C_{t}}-1\right)=k_{T}q_{e}\frac{m}{Q}-k_{T}C_{0}t$$

*C*_0_ and *C_t_* (mg/L) are the concentration of solution in the inlet and outlet at time *t* (min), *k_T_* (mL/(min·mg)) is the Thomas rate constant, *Q* (mL/min) is the flow rate, *q_e_* (mg/g) is the maximum sorption capacity, *m* (g) is the mass of activated coke.

The Yoon-Nelson model assumes that the rate of decreasing in the probability of adsorption for each adsorbate molecule is directly proportional to adsorbate breakthrough probability and adsorbate adsorption probability [41]. The Yoon-Nelson does not rely on the physical factors bed and adsorbate characteristics [42].

(4) $$\ln\left(\frac{C_{t}}{\left(C_{0}-C_{t}\right)}\right)=k_{Y}t-k_{Y}\tau$$

*C*_0_ and *C_t_* (mg/L) are the concentrations of solution in the inlet and outlet at time *t* (min), *k_Y_* (1/min) is the Yoon-Nelson rate constant, *τ* (min) is the time to reach 50% of sulfamethoxazole breakthrough. Except for the linearized Equation, Thomas and Yoon-Nelson models are equivalent in terms of mathematical form as described in Reference [43]:

(5) $$Y=\frac{1}{\left(1+\exp\left(-kt+b\right)\right)}$$

The Adams-Bohart model was based on the surface reaction theory, applied to account for the initial part of the breakthrough curve. The Adams-Bohart model and its mathematical form are as follows [40]:

(6) $$\ln\left(\frac{C_{t}}{C_{0}}\right)=K_{A}C_{0}t-N_{0}h\frac{k_{A}}{v}$$

(7) Y=exp(kt+b).

*C*_0_ and *C_t_* (mg/L) are the concentration of solution in the inlet and outlet at time *t* (min), *k_A_* (L/(min·g)) is the Yoon-Nelson rate constant, *N*_0_ (g/L) is the sorption capacity of the adsorbent per unit volume of the bed, *h* (mm) is the column height, *v* (mm/min) is the flow rate. The analysis of *C_e_*/*C*_0_ and *t* at different experimental conditions was performed. 

**Figure 7:**
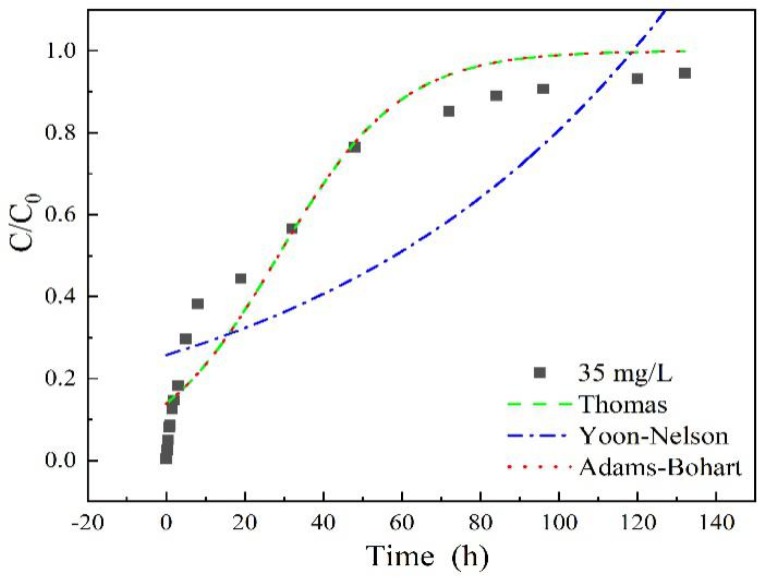
Adsorption breakthrough curves fitted by different models with sulfamethoxazole initial concentration of 35 mg/L, flow rate of 3 mL/min, pH 6.5, column height of 3 cm.

As shown in Figure 7 and Table 2, high correlation coefficients R^2^ > 0.95 were obtained in the Thomas model for all the adsorption entries (except for the column height of 7 cm), For the Yoon-Nelson model, adsorption entries under low experiment parameters (such as 35 mg/L sulfamethoxazole concentration, 3 mL/min flow rate and 7 cm column height) fitted better than the higher ones, with correlation coefficients R^2^ > 0.95. While a low R^2^ value obtained in the Adams-Bohart model indicated worse fitting results of experiment data. These calculation results demonstrated that the adsorption rate could be probably dominated by mass transfer at the interface, due to the microporous and mesoporous structure characteristics of LACs, similar conclusions were also reported in diclofenac sodium adsorption on LACs [34]. The value of the model’s parameters, such as *k_T_*, *k_Y_*, *q_e_* and *τ* depends on experimental conditions such as flow rate, initial concentration and column height. The initial concentration of sulfamethoxazole could obviously affect the maximum sorption capacity, *q_e_*, long column height of LACs may inhibit the adsorption rate due to the sharply decreasing *k_T_* in 7 cm column height entry.

### 3.3. Homogeneous Surface Diffusion Model (HDSM)

The HSDM model is one of the most widely used dual-resistance diffusion models to predict the fixed bed adsorption process. Through optimizing the fit curve to the experiment breakthrough curve, the surface diffusion coefficient *D_S_* can be obtained [44]. The HSDM was used to evaluated the diffusion coefficient of experiment data. The Equations of the HSDM model are listed as follows [45]. Freundlich isotherm parameters (*K*, 1/n) needed in HSDM model by adsorption isotherm were presented in the Supplementary material (Table S1 and Figure S1) [46].

(8) $$\frac{\partial c\left(z,t\right)}{\partial t}=D_{L}\frac{\partial^{2}c\left(z,t\right)}{\partial Z^{2}}-D\frac{\partial c\left(z,t\right)}{\partial Z}-\frac{3\left(1-\varepsilon \right)}{\varepsilon R}k_{f}\left[c\left(z,t\right)-c_{s}\left(z,t\right)\right]$$

The mass balance Equation of particle can be represented as:

(9) $$\frac{\partial q\left(r,z,t\right)}{\partial t}=D_{s}\left[\frac{\partial q^{2}\left(r,z,t\right)}{\partial r^{2}}+\frac{2}{r}\frac{\partial q\left(r,z,t\right)}{\partial r}\right]$$

The moving phase and particle concentration is correlated with Freundlich Equation:

(10) $$q\left(r=R,z,t\right)=KC_{s}{\left(z,t\right)}^{1/n}$$

The initial and boundary conditions are:

(11) $$\begin{array}{l} c\left(z=0,t>0\right)=C_{0}\\ \frac{\partial c\left(z=L,t\right)}{\partial z}=0\\ \frac{\partial q\left(r=0,z,t\right)}{\partial r}=0\\ \rho_{p}D_{s}\frac{\partial q\left(r=R,z,t\right)}{\partial r}=k_{f}\left[c\left(z,t\right)-c_{s}\left(z,t\right)\right] \end{array}$$

**Figure 8:**
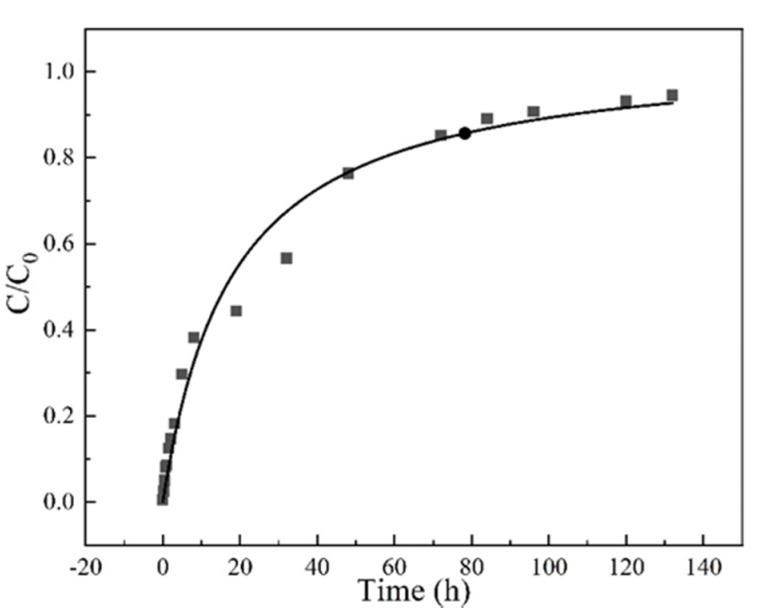
The homogeneous surface diffusion model (HDSM) diffusion model fitted breakthrough curve Experiment condition: sulfamethoxazole concentration of 35 mg/L, flow rate of 3 mL/min, pH 6.5, column height 3 cm.

The surface diffusion coefficient (*D_s_*) depends on the nature of adsorbents. So, the experimental breakthrough curves at a concentration of 35 mg/L, a flow rate at 3 mL/min, a column height of 3 cm and pH 6.5 conditions were fitted with the HSDM, as shown in Figure 8. The determined surface diffusion coefficient is 2.39 × 10^−9^ cm^2^/min.

The sum of square of error (SSE) between the experimental data and the theoretical simulation was used to evaluate the feasibility of the model. As shown in Table 3, the value of SSE is 0.04, demonstrating that the proposed HSDM model can successfully describe the adsorption and diffusion behavior in the fixed bed.

(12) $$\mathrm{SSE}={\sum \left[\frac{c_{\mathrm{cal}}-c_{\exp}}{c_{0}}\right]}^{2}$$

### 3.4. Evaluation of Factors Effect

In order to investigate the optimized parameters for dynamic adsorption of sulfamethoxazole on LACs, the effects of initial concentration, column height, flow rate, pH and organic matter on the penetration time and the length of the mass transfer zone were compared systematically. As shown in Table 4, the absolute value of average rate (Δt_b_/Δx or ΔH/Δx) were calculated from the increments of penetration time (Δt_b_), mass transfer zone length (ΔH) for each factor increments (Δx). 

The average change rates of the penetration time to the changes of adsorption parameters (Δt_b_/Δx), Δx represents initial concentration, column height, flow rate and pH, were 0.86, 98.25, 20, 12.25, respectively. Thus, column height may play a vital role in penetration time. The same results were also gained for ΔH/Δx. 

The average change rates of the length of the mass transfer zone with respect to the initial concentration, the column height, the flow rate, pH and organic matter were 0.0003, 0.6474, 0.0076, 0.0073 and 0.0191, respectively. Therefore, the change of the initial concentration has the lowest effect on the average rate of change in the length of the mass transfer zone and the column height has the greatest effect on it. These calculations were in accordance with the studies in the above adsorption effects sections.

## 4. Conclusions

Dynamic column adsorption of sulfamethoxazole by lignite activated coke from aqueous solution were conducted under different initial sulfamethoxazole concentrations, flow rates, column height and pH to investigate the optimized adsorption conditions for wastewater treatment. Results demonstrated that activated coke has high adsorption efficiency for sulfamethoxazole removal from aqueous solution. Increasing the concentration of pollutants and the flow rate will shorten the penetration time, while opposite tendency was found in the column height effect. The best adsorption performance was obtained at pH 6.5 due to the possible hydrogen bonding interaction between sulfamethoxazole species and LACs. The adsorption capacity of activated coke increased by increase in the column height. The Thomas model could best describe the column adsorption behavior with R^2^ > 0.95 for nearly all adsorption entries. The column height of the activated cokes played a vital role in long penetration time and mass transfer zone.

## Figures and Tables

**Table 1:** Comparative data of penetration curves of dynamic coke adsorption sulfamethoxazole dynamic column experiments.

Concentration (mg/L)	Flow Rate (mL/min)	Column Height (cm)	pH	Humic Acids (mg/L)	10% Breakthrough Volume (cm^3^)	95% Breakthrough Volume (cm^3^)
35	3	3	6.5	0	210	23745
70	3	3	6.5	0	110	20820
35	3	3	6.5	0	210	23745
35	5	3	6.5	0	98	24000
35	3	3	6.5	0	210	23745
35	3	7	6.5	0	395	30000
35	3	3	4	0	15	20700
35	3	3	6.5	0	210	23745
35	3	3	8	0	115	12636
35	3	3	6.5	0	210	23745
35	3	3	6.5	0.1	680	-
35	3	3	6.5	1	20	17220
35	3	3	6.5	10	250	26190

**Table 2:** The summary of experiment parameters calculated from different models under various experimental conditions.

Models	Initial Concentration (mg/L)		Flow Rate (mL/min)		Column Height (cm)	
	35	70	3	5	3	7
Thomas						
R^2^	0.953	0.951	0.953	0.950	0.953	0.93
q_e_ (mg/g)	3.04	11.56	3.04	4.37	3.04	3.09
k_T_ (mL/(mg·min))	0.039	0.032	0.039	0.039	0.039	0.010
Yoon-Nelson						
R^2^	0.95	0.90	0.953	0.91	0.953	0.93
τ (h)	25	12	25	12	38.7	80
k_Y_ (1/min)	0.045	0.039	0.045	0.078	0.045	0.038
Adams-Bohart						
R^2^	0.72	0.63	0.72	0.63	0.72	0.71
k_A_ (L/(mg·min))	0.456	0.375	0.456	0.377	0.456	0.54
N_0_ (g/L)	2.6	3.0	2.6	2.5	2.6	2.83

**Table 3:** The SSE of HSDM models.

**Time (h)**	0	0.25	0.5	0.75	1	1.5	2	3	5
**C_cal_**	0.00	0.01	0.03	0.04	0.06	0.08	0.11	0.15	0.23
**C_exp_**	0.00	0.03	0.05	0.08	0.09	0.13	0.15	0.18	0.30
**Time (h)**	8	19	32	48	72	84	96	120	132
**C_cal_**	0.32	0.54	0.67	0.77	0.84	0.87	0.89	0.92	0.93
**C_exp_**	0.38	0.44	0.57	0.76	0.85	0.89	0.91	0.93	0.95
**SSE**	0.04								

**Table 4:** Analysis of each factor on the length of the mass transfer zone of the activated coke adsorption sulfamethoxazole dynamic column experiment.

Factor	Variable X	H	Δx	Δt_b_	ΔH	|Δt_b_/Δx|	|ΔH/Δx|
Initial sulfamethoxazole concentration	35 mg/L	2.973	35	30	0.009	0.86	0.003
	70 mg/L	2.982					
Column height	3 cm	2.988	4	393	2.59	98.25	0.6474
	7 cm	5.578					
Flow rate	3 mL/min	2.973	2	40	0.015	20	0.0076
	5 mL/min	2.988					
pH	4	3.346	4	61	0.448	12.25	0.0073
	6.5	2.973					
	8	3.421					
Humic acids	0.1 mg/L	3.474	9.9	220	0.189	2.22	0.0191
	1 mg/L	3.333					
	10 mg/L	3.285					

Note: t_b_ is the penetration time; t_e_ is the end time of penetration; H is the length of the mass transfer zone; Δx = x_max_−x_min_; Δt_b_ = t_bmax_−t_bmin_; ΔH = H_max_−H_min_

## Supplementary Materials

The following are available online at https://www.mdpi.com/1996-1944/13/7/1785/s1, Figure S1: Adsorption isotherms of sulfamethoxazole. Condition: under low initial concentrations of 1~50 mg/L. Adsorbent dosage = 0.5 g/L, pH = 6.5 ± 0.1, [NaCl] = 10 mmol/L, Table S1: Adsorption isotherms parameters.
