# Supplementary material for: Dynamic Adsorption of Sulfamethoxazole from Aqueous Solution by Lignite Activated Coke
Source: Materials (Basel). 2020 Apr 10;13(7):1785. doi: 10.3390/ma13071785 (PMC7179028; doi:10.3390/ma13071785)
Supplement: Supplementary file 1 [file materials-13-01785-s001.pdf]

materials

Supplementary material

Dynamic Adsorption of Sulfamethoxazole from Aqueous Solution by Lignite Activated Coke

Haiyan Li [1,2], Juan He [1], Kaiyu Chen [1], Zhou Shi [1], Mengnan Li [1], Pengpeng Guo [1] and Liyuan Wu [1,2,*]

1 Beijing Engineering Research Center of Sustainable Urban Sewage System Construction and Risk Control, Beijing University of Civil Engineering and Architecture, 100044 Beijing, China; Lihaiyan@bucea.edu.cn (H.L.), louyounan@163.com (J.H.); Chenkaiyuxiao@163.com (K.C.); shizhou2016@163.com (Z.S.); lmn970109@163.com (M.L.); gzpjustice@163.com (P.G.)

2 Beijing Advanced Innovation Center for Future Urban Design, Beijing 100044, China.

* Correspondence: wuliyuan@bucea.edu.cn; Tel.: +86 010 68322452

Received: 2 March 2020; Accepted: 7 April 2020; Published: date

1. Adsorption Isotherms

The Freundlich model [1] was as follows:

$$q_e = FC_e^n \quad (1)$$

where q_e (mg/g) and C_e (mg/L) are the adsorption capacity and the concentration of sulfamethoxazole at equilibrium, respectively; F, n are the empirical constants.

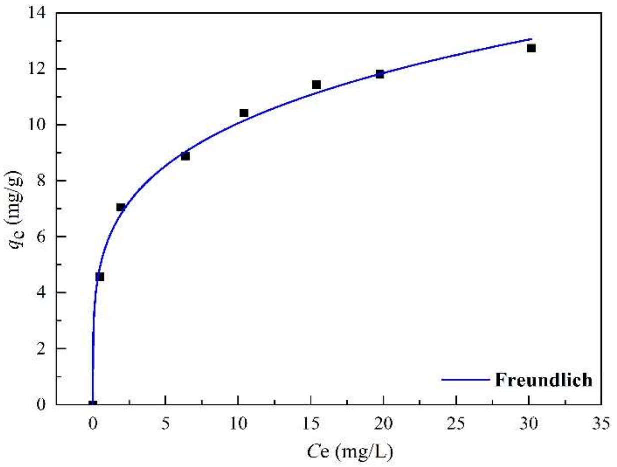

Figure S1. Adsorption isotherms of sulfamethoxazole. Condition: under low initial concentrations of 1~50 mg/L. Adsorbent dosage = 0.5 g/L, pH = 6.5 ± 0.1, [NaCl] = 10 mmol/L.

Table S1. Adsorption isotherms parameters.

Model	Parameters	Value
	K	5.85
**Freundlich**	1/n	0.23
	R^2	0.996

References

1. Li, Z.; Wu, L.; Liu, H.; Lan, H.; Qu, J. Improvement of aqueous mercury adsorption on activated coke by thiol-functionalization. *Chem. Eng. J.* **2013,** *228*, 925–934.
